# Effects of *Acanthus ebracteatus* Vahl. extract and verbascoside on human dermal papilla and murine macrophage

**DOI:** 10.1038/s41598-022-04966-w

**Published:** 2022-01-27

**Authors:** Vanuchawan Wisuitiprot, Kornkanok Ingkaninan, Panlop Chakkavittumrong, Wudtichai Wisuitiprot, Nitra Neungchamnong, Ruttanaporn Chantakul, Neti Waranuch

**Affiliations:** 1 Department of Pharmaceutical Technology, Faculty of Pharmaceutical Sciences and Center of Excellence for Innovation in Chemistry, Naresuan University, Phitsanulok, 65000 Thailand; 2 Department of Pharmaceutical Chemistry and Pharmacognosy, Faculty of Pharmaceutical Sciences, Naresuan University, Phitsanulok, 65000 Thailand; 3 Division of Dermatology, Department of Medicine, Faculty of Medicine, Thammasat University, Khlong Luang, Pathumthani 12121 Thailand; 4 Department of Thai Traditional Medicine, Sirindhorn College of Public Health, Phitsanulok, 65130 Thailand; 5 Science Laboratory Centre, Faculty of Science, Naresuan University, Mueang, Phitsanulok, 65000 Thailand; 6 Bioscreening Unit, Department of Pharmaceutical Chemistry and Pharmacognosy, Faculty of Pharmaceutical Sciences, Naresuan University, Phitsanulok, 65000 Thailand; 7 Cosmetics and Natural Products Research Center, Faculty of Pharmaceutical Sciences, Naresuan University, Phitsanulok, 65000 Thailand

**Keywords:** Drug discovery, Biomarkers, Health care, Medical research

## Abstract

Androgenic alopecia is a common type of hair loss, usually caused by testosterone metabolism generating dihydrotestosterone and hair follicular micro-inflammation. These processes induce dermal papilla cells to undergo apoptosis. Currently approved effective medications for alopecia are Finasteride, an oral 5α-reductase inhibitor, Minoxidil, a topical hair growth promoter, and Diclofenac, an anti-inflammatory agent, all of which, however, have several adverse side effects. In our study, we showed the bioactivity of *Acanthus ebracteatus* Vahl. (AE) extract performed by 95% ethanol, and verbascoside (VB), a biomarker of AE extract. Both AE extract and VB were studied for their effects on dermal papilla cell viability and the cell cycle by using MTT assay and flow cytometry. The effect of an anti-inflammatory activity of AE extract and VB on IL-1β, NO, and TNF-α, released from LPS induced RAW 264.7 cells, and IL-1α and IL-6 released from irradiated dermal papilla cells were detected using ELISA technique. The preventive effect on dermal papilla cell apoptosis induced by testosterone was determined by MTT assay. In controlled in vitro assays it was found that AE extract and VB at various concentrations induced dermal papilla cell proliferation which was indicated by an increase in the number of cells in the S and G2/M phases of the cell cycle. AE extract at 250 µg/mL concentration or VB at 62.50 µg/mL concentration prevented cell apoptosis induced by testosterone at a statistically significant level. In addition, both AE extract and VB greatly inhibited the release of pro-inflammatory cytokines from RAW 264.7 and dermal papilla cells. The release of IL-1β, TNF-α, and NO from RAW 264.7 cells, as well as IL-1α and IL-6 from dermal papilla cells, was also diminished by AE extract 250 µg/mL and VB 125 µg/mL. Our results indicate that AE extract and VB are promising ingredients for anti-hair loss applications. However, further clinical study is necessary to evaluate the effectiveness of AE extract and VB as treatment for actual hair loss.

## Introduction

Androgenic alopecia (AGA) is the most common form of hair loss in men and women and can be serious enough to have a detrimental effect on an individual’s social life and self-confidence. Several hypotheses regarding the mechanism and cause of hair loss have suggested that the most influential factor in hair loss is the metabolism of testosterone into the more potent androgen dihydrotestosterone (DHT) using enzyme 5 α-reductase. DHT then binds with the androgen receptor in the cytosol of dermal papilla cells to form a complex. The androgen receptor complex translocate into the nucleus and consequently activates androgen receptor-regulated genes^1^. This results in stimulation of TGF-beta mRNA synthesis in the dermal papilla cells. TGF-β is an important trigger of the intrinsic caspase network and programmed cell death, or apoptosis, of derma papilla cells^2^. The apoptosis of dermal papilla cells also induces the catagen phase of the hair growth cycle, causing the hair to stop growing^3,4^.

Another contributing factor to hair loss is hair follicle microinflammation which has been identified in patients presenting baldness^1^. Researchers have noted that some inflammatory cytokines in alopecia patients are higher than in the unaffected scalp. Others reported that inflammatory cytokines, such as IL-1β, IL-1α, IL-6, TNF- α, NO and prostaglandin D2, destroyed hair follicle structure and spurred dermal papilla to undergo apoptosis^5–9^. Inflammation and the apoptosis of cells involve the caspase-1 named inflammasome, which triggers an inflammatory chain reaction. Testosterone and DHT have also been found to contribute to inflammation^10^. According to two influential factors mentioned previously, anti-testosterone activity has been concerned particularly with 5α-reductase inhibition activity.

Anti-inflammatory agents are often chosen for multimodal treatments for alopecia^11–16^. However, many of these treatments have undesirable side effects. Finasteride is a synthetic 5α-reductase inhibitor combined with an anti-inflammatory agent that is used to treat androgenic alopecia induced by the androgenic hormones^17^. Although finasteride is an effective treatment for AGA, patients taking this medication also suffer the detrimental side effect of sexual dysfunction. Moreover, finasteride is not approved for use in women with hair loss, particularly as it is especially dangerous for pregnant women, possibly causing abnormalities of the external genitalia in the male fetus^13^. Anti-inflammatory drugs in the NSAIDs group, used as a hair loss treatment, have diverse side effects such as GI disturbance and the potential negative effects during pregnancy^18,19^. Minoxidil is also a common treatment for alopecia^13,20^. However, common side effects of topical minoxidil administration are facial hypertrichosis and local intravascular spread of minoxidil. In some cases, patients using minoxidil have allergic reactions to propylene glycol, which is used in commercial minoxidil preparation^21,22^.

There is, therefore, a need to develop new hair loss applications because of these various adverse side effects of current hair loss medications. Due to a growing understanding of the mechanisms causing AGA, researchers are interested in investigating herb-derived compounds that show anti-androgenic and anti-inflammatory activity as potential new ingredients for hair loss treatments.

*Acanthus ebracteatus* Vahl. (AE) is a medicinal plant that has been traditionally used as an anti-inflammatory agent, and has been used in traditional medicines as an ingredient in anti-cancer and anti-inflammatory formulations^23^. One report from Malaysia recommends that the leaves of AE be used as an anti-hair loss remedy^24^. A few reports have found that the ethanolic extract of AE contained various bioactive compounds including verbascoside (VB)^25^, which is a promising phenylpropanoid compound in phytotherapy. Other studies have reported the anti-inflammatory activity of VB that has been isolated from many local herbs. Cyclooxygenase-1 (COX-1) and Cyclooxygenase-2 (COX-2) were inhibited by VB isolated from *Marrubium vulgare* with IC_50_ at 1.00 mM and 0.69 mM concentrations, respectively^26^. One article reported that VB decreased the expression of the COX enzyme by increasing the phosphorylation of the Src homology region 2 domain-containing phosphatase-1 (SHP-1). SHP-1 plays a crucial role in the synthesis of COX-2 and inducible nitric oxide synthase (iNOS)^27^. In addition, recent studies reported that the anti-inflammatory activity of VB was due to the suppression of TGF-β gene expression^27,28^. VB also affects the testosterone metabolism in Leydig cells and also decreases the testosterone level by down-regulating cAMP and preventing cell apoptosis^29^. Thus, VB might be a promising compound for treating hair loss induced by testosterone metabolism and hair follicle inflammation. The objectives of our study were to identify the bioactivity of AE extract and VB, particularly their potential anti-androgenic and anti-inflammatory activities.

We investigated the inhibitory effect of AE extract and VB against the factors that contribute to hair loss in the human dermal papilla and mouse macrophage cells. Specifically, we examined 5α-reductase inhibition activity, anti-inflammatory actions through the inhibition of IL-1β, IL-1α, TNF-α, NO, IL-6 expression, and protection against dermal papilla apoptosis.

## Results

### Plant extract preparation and VB analyzed using HPLC

**Figure 1:**
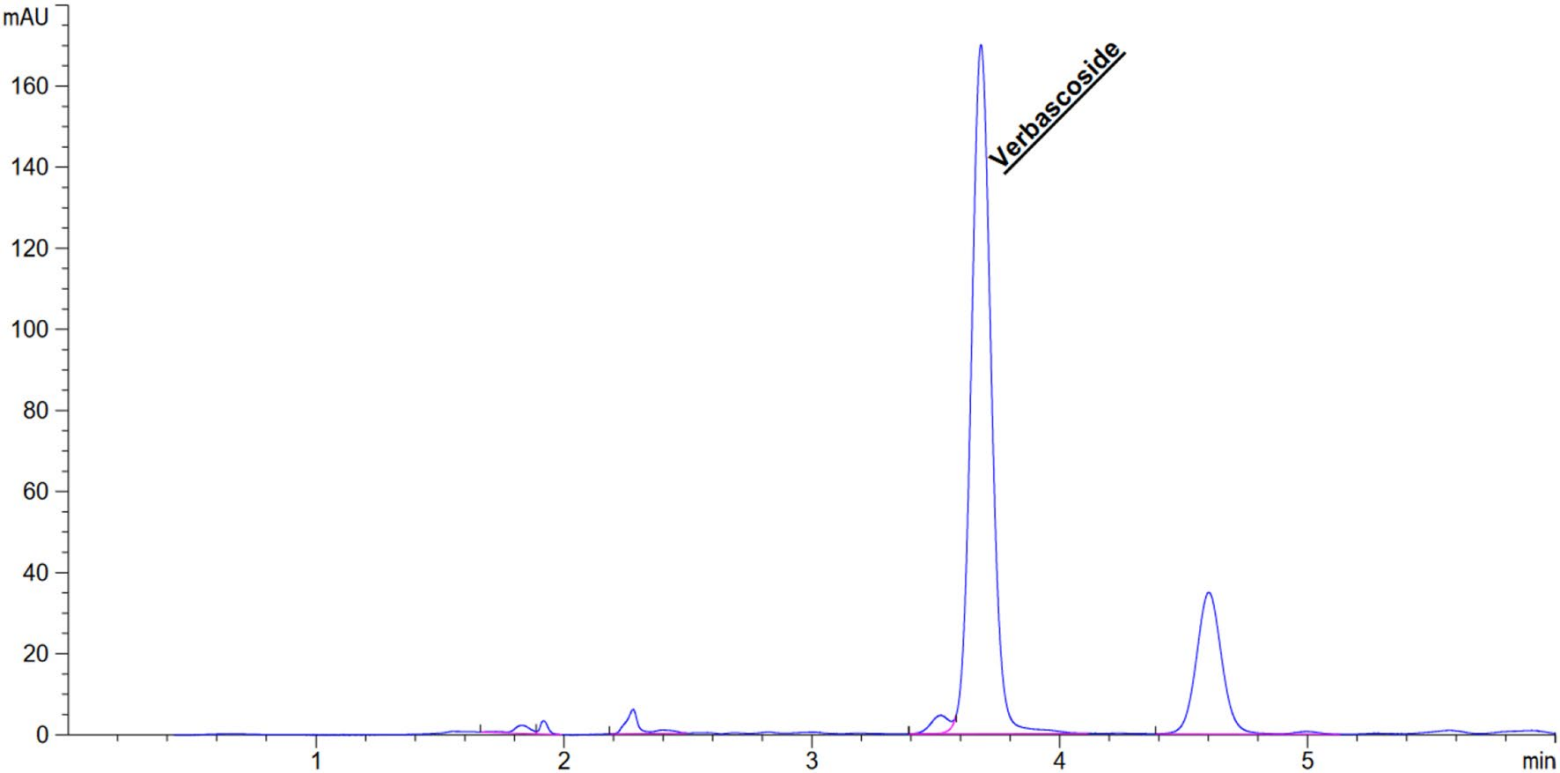
Sample high-performance liquid chromatography (HPLC) chromatogram of VB in 95% ethanol extract of AE.

AE extracts were prepared using 95% ethanol, 50% ethanol, water and hexane. The amount of VB in each extract was measured using the HPLC technique. The indicative HPLC method linearity was defined as having r^2^ greater or equal to 0.9997 using concentrations ranging from 19.53 to 1000.00 µg/mL. The limit of detection (LOD) value of VB was 0.04 pg/mL while the limit of quantification (LOQ) was 0.12 pg/mL. This method was precise with intra-day relative standard deviation (%RSD) less than 5.84% and inter-day values less than 5.05%. The method also showed high accuracy with percentage recovery ranging from 90 to 110%. Therefore, this HPLC method was accurate and reliable for the determination of the amount of VB in AE extract. Sample chromatogram of VB in AE extract with 95% ethanol is presented in Fig. 1.

VB contents in various AE extracts are shown in Table 1. The highest content of VB was found in an extract using 95% ethanol (9.58 ± 0.65%). In contrast, VB could not be detected in the AE extract using hexane. These results suggest that AE extract in 95% ethanol was a promising candidate for further study because it had the highest content of VB.

**Table 1:** Amount of VB in AE extracts obtained with various solvents, measured by high-performance liquid chromatography (HPLC) (n ≥ 3).

Type of solvent for AE extract	VB (% ± SD, w/w)
95% ethanol extract	9.58 ± 0.65
50% ethanol extract	2.03 ± 0.04
Water extract	3.10 ± 0.10
Hexane extract	Below limit of detection

### Determination of 5α-Reductase Inhibition

One hundred micrograms per milliliter of each AE extract was determined for 5α-reductase inhibitory activity. Finasteride was used as the positive control. Their inhibitory activities are shown in Table 2. The comparison of the inhibition activities indicated that AE extract with 95% ethanol showed the highest inhibition (38.26 ± 4.90%), followed by AE extract with hexane (36.92 ± 2.41%), 50% ethanol (13.72 ± 4.88%) and water (6.75 ± 4.20%) while, surprisingly, no inhibitory activity was found for VB. AE extract with 95% ethanol was selected for further bioactivity studies of both VB content and 5α-reductase inhibitory activity.

**Table 2:** 5α-reductase inhibitory activity of Finasteride, VB and AE extracts of various solvents. (n ≥ 3).

Type of sample	5α-reductase inhibition (% ± SD)
AE extract in 95% ethanol	38.26 ± 4.90
AE extract in 50% ethanol	13.72 ± 4.88
AE extract in water	6.75 ± 4.20
AE extract in hexane	36.92 ± 2.41
VB	None
Finasteride	98.58 ± 1.96

AE extract and VB concentrations were 100 µg/mL, finasteride concentration was 1.5 μg/mL.

### Cytotoxic effect of AE extract and VB on human dermal papilla cells

**Figure 2:**
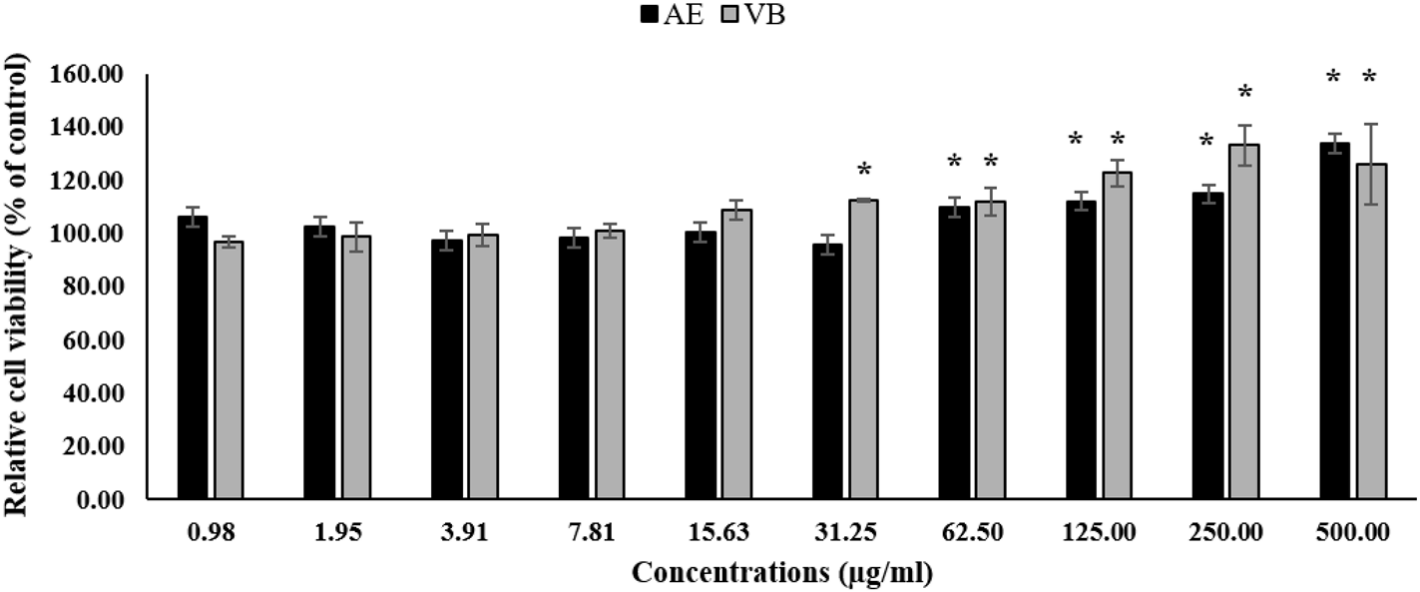
Relative cell viability of dermal papilla cells treated with AE extract or VB for 24 h (* significantly different from control cells at *p* < 0.05, Student’s *t*-test). (n ≥ 3).

The cytotoxic effect of AE extract with 95% ethanol on dermal papilla cells was evaluated by MTT assay. The results indicated that AE extract and VB at a concentration of 0.98 to 500 µg/mL did not show any cytotoxic effects on dermal papilla cells (Fig. 2). All of the treated cells presented relative cell viability of more than 80% compared to the control cells^30^. Interestingly, VB at 31.25 to 500 µg/mL concentration and AE extract at 62.50 to 500 µg/mL concentration showed significantly increased relative cell viability of dermal papilla cells. This means that AE extract and VB at these concentrations might be able to activate dermal papilla cell proliferation.

### Cell cycle analysis

**Figure 3:**
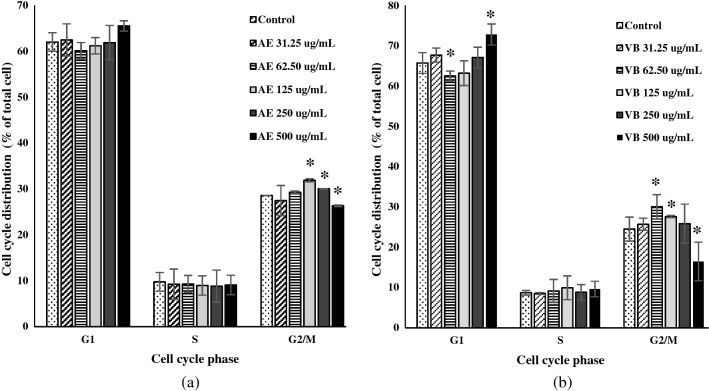
The effects of AE extract (**a**), VB (**b**) on the dermal papilla cell cycle; * significantly different from control at *p* < 0.05 Student’s *t*-test. (n ≥ 3).

Dermal papilla cells were treated with AE extract of 95% ethanol or VB at a concentration between 31.25 and 500 µg/mL for 24 h. They were then analyzed at different points in the cell cycle with propidium iodide staining. AE extract showed different effects on the dermal papilla depending on concentrations (Fig. 3a). The dermal papilla cell growth (G1 phase) was stimulated by AE extract 500 µg/mL. However, the preparation for cell division (G2/M phase) was lower than that of the control cells. On the contrary, AE extract at 250 µg/mL and 125 µg/mL concentrations showed a significantly increased number of cells in the G2/M phase compared to the control cells. In the case of VB, the concentration of 500 µg/mL significantly increased the number of cells in the G1 phase (Fig. 3b), but decreased the number of cells in the G2/M phase compared to the control cells. The positive effects of VB 62.50 µg/mL on the G2/M and G1 phases of the dermal papilla cells were significant. The number of cells in the G2/M phase significantly increased, while the number of cells in the G1 phase decreased after being treated by VB 62.50 µg/mL. In addition, treating the cells with VB 125 µg/mL resulted in an increased number of cells in the G2/M phase compared to the control cells while no difference of G1 phase from control cells was observed. All of the treatment concentrations had no effects on the number of cells in the DNA replication stage (S phase) of the cell cycle.

### Inhibition effects of AE extract and VB on testosterone-induced cell apoptosis

**Figure 4:**
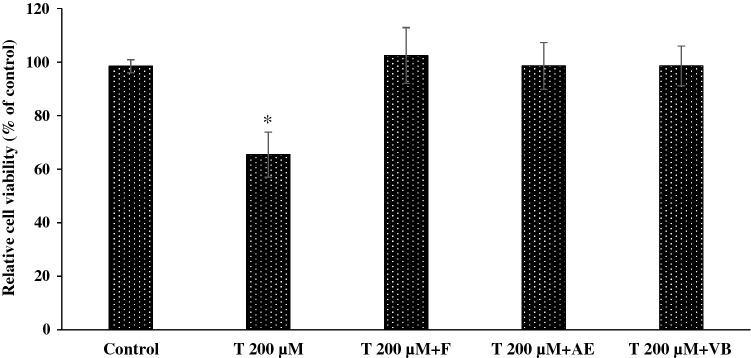
Relative cell viability of dermal papilla cells when being treated with testosterone 200 µM (T 200 µM), testosterone 200 µM plus AE extract 250 µg/ml (T 200 µM + AE), testosterone 200 µM plus finasteride 75 nM (T 200 µM + F) and testosterone 200 µM plus VB 62.50 µg/mL (T 200 µM + VB) (* significantly different from control at *p* < 0.05, Student’s *t*-test). (n ≥ 3).

Cell apoptosis was determined in terms of cell viability. The concentration of 250 µg/mL 95% ethanol AE extract was the optimal value identified in our cell cycle analysis and was therefore selected for these tests. Two hundred micromolar concentrations of testosterone caused a decrease in dermal papilla cells viability when the cells were treated for 4 days (Fig. 4). Cell viability remained close to 100% when the dermal papilla cells were treated with 200 µM testosterone and 250 µg/mL AE extract in combination. VB 62.50 µg/mL was also effective in preventing testosterone induced cell apoptosis, showing results similar to AE extract 250 µg/mL or 75 nM finasteride.

### Cytotoxic effect of AE extract and VB on murine macrophage RAW 264.7 cells

**Figure 5:**
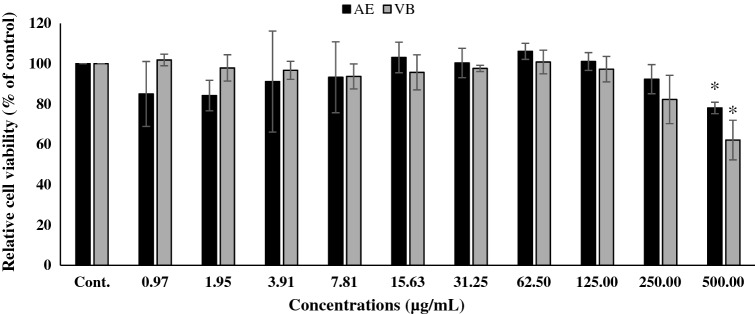
Relative cell viability of RAW 264.7 cells treated with different concentrations of AE extract or VB for 24 h; Cont. = Control. * Significantly different from control at *p* < 0.05 Student’s *t*-test. (n ≥ 3).

Cytotoxicity of various concentrations of 95% ethanol AE extract and VB on RAW 264.7 cells was determined using MTT assay. The results are shown in Fig. 5. The study found that both AE extract and VB were safe at concentrations up to 250 µg/mL. Thus, we selected these as the upper limit concentrations for further study on anti-inflammatory activity in macrophage cells.

### Effects of AE extract and VB on IL-1β, NO, and TNF-α production in LPS-stimulated macrophages

**Figure 6:**
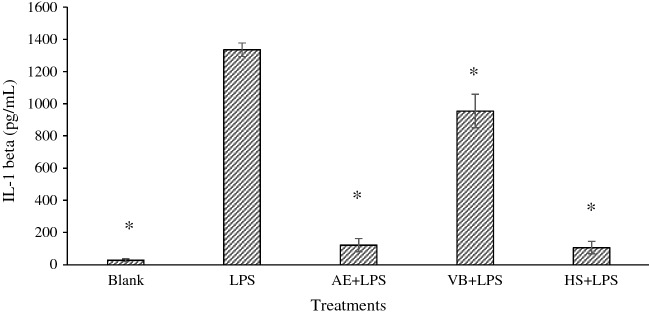
Amount of IL-1β from RAW 264.7 cells treated with lipopolysaccharide 5 µg/mL (LPS), AE extract 250 µg/mL plus LPS 5 µg/mL (AE + LPS), Hydrocortisone 5 µg/mL plus LPS 5 µg/mL (HS + LPS) and VB 125 µg/mL plus LPS 5 µg/mL (VB + LPS); * significantly different from LPS at *p* < 0.05 Student’s *t*-test. (n ≥ 3).

**Figure 7:**
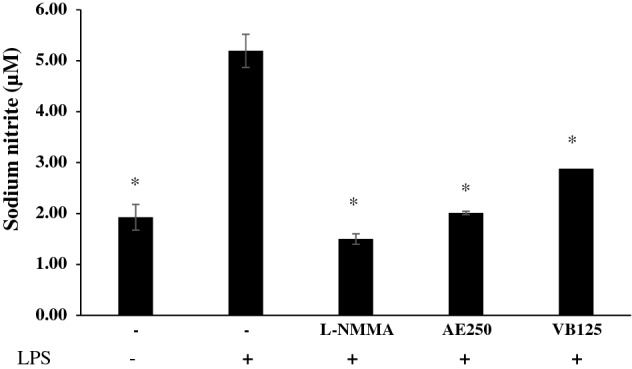
Amount of sodium nitrite from RAW 264.7 cells treated by lipopolysaccharide 1 µg/mL (LPS), AE extract 250 µg/mL plus LPS (AE + LPS), N(G)-monomethyl-l-arginine 10 µg/mL (L-NMMA) plus LPS (L-NMMA + LPS), and VB 125 µg/mL plus LPS (VB + LPS); * significantly different from LPS treated RAW 264.7 cell at *p* < 0.05 Student’s *t*-test. (n ≥ 3).

**Figure 8:**
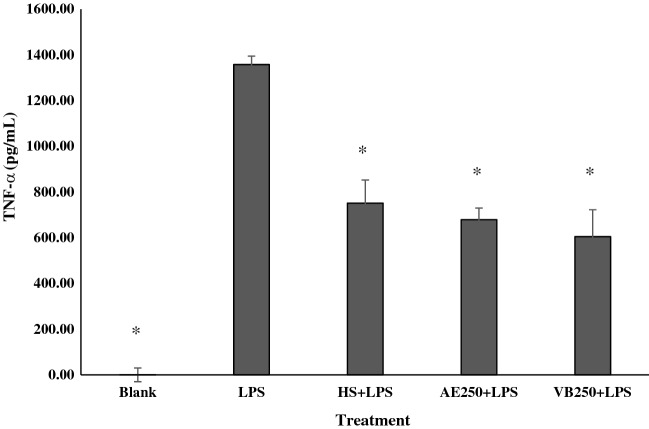
Amount of tumor necrosis factor (TNF-α) from RAW 264.7 cells treated with lipopolysaccharide 1 µg/mL (LPS), hydrocortisone 10 µg/mL + LPS 1 µg/mL (HS + LPS), AE extract 250 µg/mL plus LPS 1 µg/mL (AE250 + LPS) and VB 250 µg/mL plus 1 µg/mL LPS (VB250 + LPS); * significantly different from LPS treated RAW 264.7 cell at *p* < 0.05 Student’s *t*-test. (n ≥ 3).

RAW 264.7 cells were treated by LPS to induce the release of IL-1β, NO and TNF-α, and these cytokines were detected using the ELISA technique. AE ethanolic extract 250 µg/mL or VB 125 µg/mL was selected for IL-1β and NO testing, while both AE extract and VB at 250 µg/mL were used for TNF-α determination. The different concentrations used in this study were taken from the potency screening previously undertaken in our previous work (data not shown). Hydrocortisone was a positive control. The amount of cytokines released from macrophage cells is shown in Figs. 6, 7 and 8.

LPS activated pro-inflammatory cytokines such as IL-1β, NO and TNF-α were released from RAW 264.7 cells. Those cytokines can be inhibited by steroid agents such as hydrocortisone, a synthetic preparation of the steroid hormone cortisol, which has an anti-inflammatory response^31–33^. Like hydrocortisone 5 µg/mL, AE extract 250 µg/mL showed successful downregulation of IL-1β released from the RAW 264.7 cells treated with LPS (Fig. 6). Similarly, there was also significant inhibition but to a lesser degree when VB 125 µg/mL was used.

RAW 264.7 cells treated with LPS also produced nitric oxide, which was represented by sodium nitrite content. N(G)-monomethyl-l-arginine (L-NMMA) was used as a positive control. As shown in Fig. 7, the results were that LPS effectively induced the synthesis of sodium nitrite in Raw 264.7 cells. L-NMMA inhibited the synthesis of NO activated by LPS due to the inhibition of nitric oxide synthase (NOS)^34^. Both AE extract 250 µg/mL and VB 125 µg/mL also showed greater potency of inhibition of nitric oxide secretion than the LPS control cells.

Tumor necrosis factor (TNF-α) released from LPS treated macrophage cells was strongly inhibited by hydrocortisone (Fig. 8). Similar to hydrocortisone, 250 μg/mL AE extract and 250 μg/mL VB also significantly suppressed the release of TNF-α.

### Effects of AE extract and VB on IL-1α and IL-6 production in UVB irradiated dermal papilla cells

**Figure 9:**
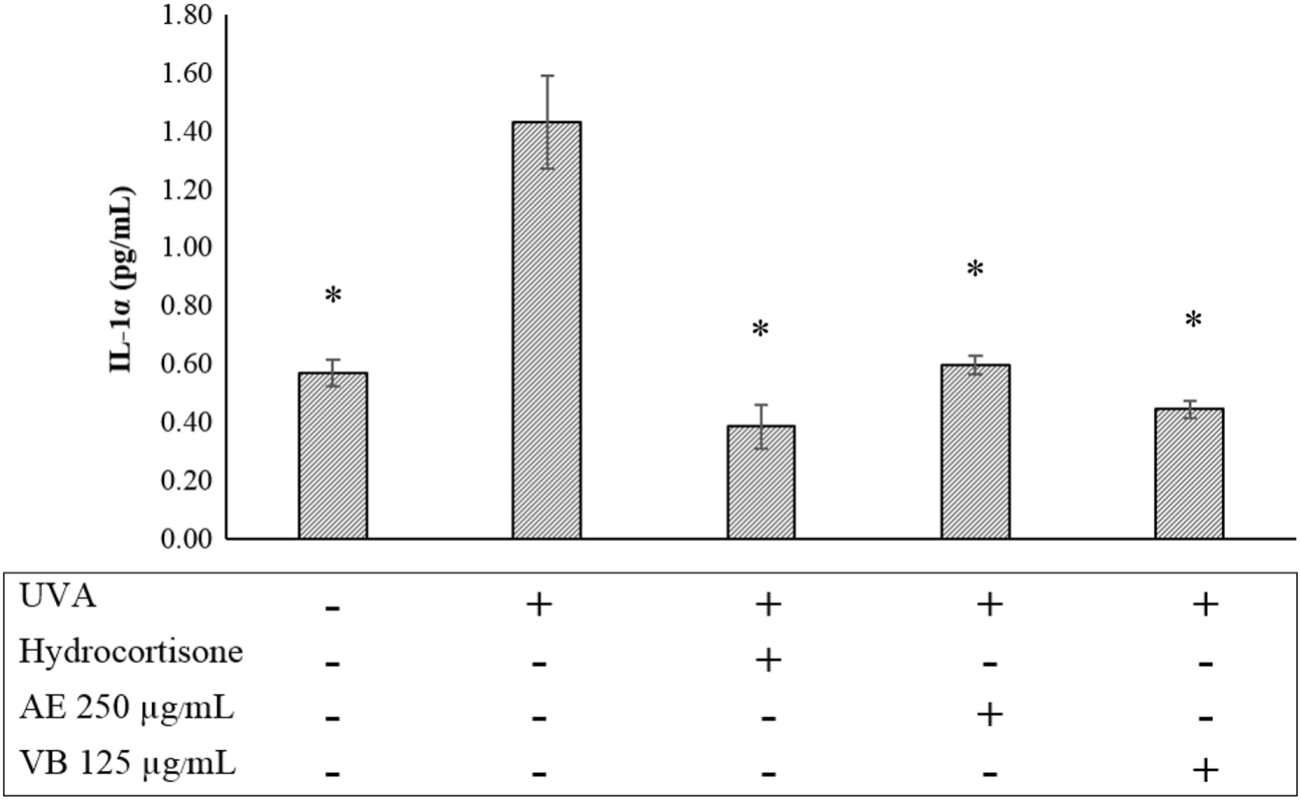
The release of IL-1α from dermal papilla cells following UVA 20 mJ/cm^2^ irradiation and after treatment with hydrocortisone 5 µg/mL, AE extract 250 µg/mL and VB 125 µg /mL; * significantly different from irradiated cells at *p* < 0.05 Student’s t-test. (n ≥ 3).

**Figure 10:**
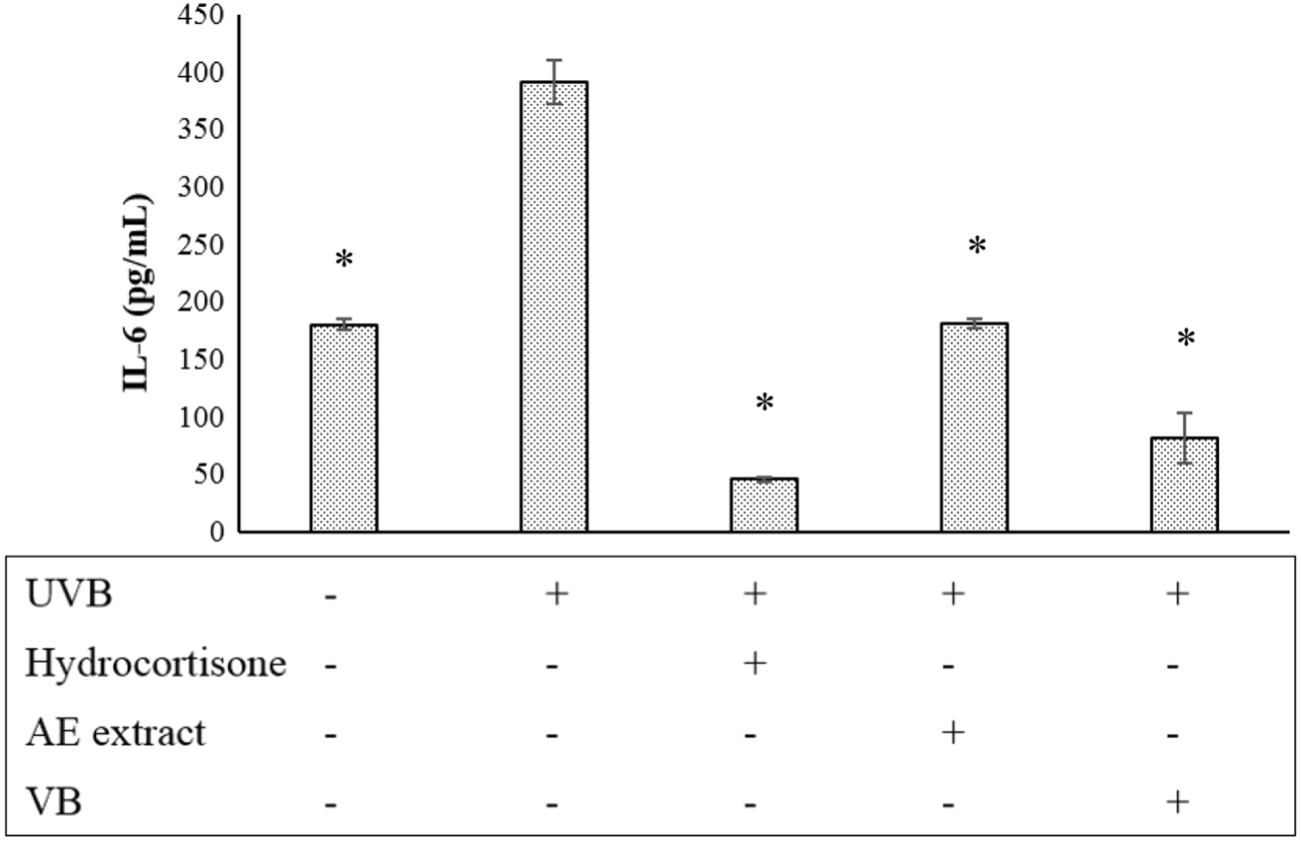
The release of IL-6 from dermal papilla cells following UVB 100 mJ/cm^2^ irradiation and irradiated cell after treatment with hydrocortisone 5 µg/mL, AE extract 250 µg/mL and VB (VB) 125 µg/mL; * significantly different from irradiated cells at *p* < 0.05 Student’s *t*-test. (n ≥ 3).

Dermal papilla cells released IL-1α and IL-6 in the supernatant fluid after being irradiated with 20 J/cm^2^ UVA and 100 mJ/cm^2^ UVB, respectively. IL-6 and IL-1α levels in the supernatant were measured using cytokine immunoassay (ELISA technique). Hydrocortisone 5 µg/mL was a positive control. The amount of IL-1α is shown in Fig. 9, and the amount of IL-6 is shown in Fig. 10.

Figure 9 shows that AE extract 250 µg/mL or VB 125 µg/mL significantly decreased the amount of IL-1α released from the irradiated dermal papilla cells. Similar to IL-1α, AE extract 250 µg/mL and VB 125 µg/mL significantly suppressed the release of IL-6 (Fig. 10).

## Discussion

The bioactivity of AE extract and VB on murine macrophage RAW 267.4 and human dermal papilla cells was evaluated. The RAW 264.7 cells are normally used for evaluating anti-inflammatory activity because the macrophage can be stimulated by LPS to produce IL-1β by activating Toll-like receptors^35,36^. Although the release of pro-inflammatory cytokines in dermal papilla cells has been not fully understood, many reports also reveal the determination of those cytokines from dermal papilla cells and they play a crucial role in dermal papilla cell apoptosis and hair follicular damage^1,8,9,37^. AE extracted with 95% ethanol had the highest content of VB of all tested AE extracts. Both AE extracted with 95% ethanol and VB were non-toxic to the dermal papilla and macrophage cells in the concentration range 0.98 to 500 µg/mL. The inhibition activity of 5α-reductase was evaluated by using an enzymatic activity assay. The enzyme used was isolated from LNCaP cell that presents the prominent quantity in 5α-reductase^38,39^. AE extracted with 95% ethanol possessed the highest 5α-reductase inhibition activity (38.26 ± 4.90% at 100 µg/mL). Surprisingly, this activity was not found for VB. These results indicate that other components than VB in the AE extract demonstrate 5α-reductase inhibition activity. Although VB did not appear to affect 5α-reductase inhibition, VB 62.50 µg/mL inhibited dermal papilla cell apoptosis induced with 200 µM testosterone. Similar activity was also found with 250 µg/mL AE extracted with 95% ethanol and 75 nM finasteride. This effect could be due to the down-regulation of cAMP, which prevents cell apoptosis induced by testosterone^29^. AE extract and VB did not show any cytotoxic effects on dermal papilla cells, but these compounds did induce dermal papilla cell proliferation.

Cell cycle study indicated that both AE extract and VB at the concentration of 500 µg/mL suppressed dermal papilla cell proliferation (G2/M phase). The increasing numbers of cells in the G1 phase were evidenced for 500 µg/mL AE extract and 500 µg/mL VB, which indicated the induction of G1 phase of dermal papilla cell cycle arrest^40,41^. However, AE extract (125 µg/mL and 250 µg/mL) and VB (62.50 µg/mL and 125 µg/mL) promoted dermal papilla cell proliferation. At these concentrations, the cell numbers in the G2/M or S phases were greatly increased compared to control cells. The increased numbers of cells in the G2/M or S phases in the cell cycle indicated that the cells were in the process of DNA synthesis and the proliferation stages^42,43^. Although 125 μg/mL and 250 μ/mL of AE extracts did not show any effect on the G1 phase of the cell cycle, 62.50 μg/mL of VB significantly decreased the cell number in the G1 phase when compared to control cells. The decrease of cell number in the G1 phase has implied the increase of cell proliferation^42^. Those results were consistent with the results of anti-testosterone activity tests that showed that AE extracts (250 µg/mL) and VB (62.50 µg/mL) prevented cell apoptosis induced by testosterone. The human dermal papilla cell apoptosis induced by testosterone have been well documented^44^. The prevention of cell apoptosis via down-regulating cAMP was reported as a mechanism of VB on testosterone activity^29^. The decrease in testosterone metabolism diminished inflammation initiating cell apoptosis^1,9^.

IL-1β and IL-1α are pro-inflammatory cytokines that indicate cells are undergoing an inflammatory process^45^. In various cell types, the IL-1 family can be induced to release several inflammatory cytokines, such as IL-6, prostaglandin E2, tumor necrotic factor-alpha (TNF-α), and nitric oxide (NO)^36,46,47^. The release of NO can be detected by measuring nitrate and nitrite levels in cell culture supernatants. NO is a signaling molecule in the hair follicle. Overexpression of NO is involved in the regulation and differentiation of hair follicles resulting in hair loss^5^. RAW 264.7 cells were used to induce IL-1β, TNF-α and NO-releasing by LPS^35,48^. AE extract 250 µg/mL and VB 125 µg/mL were observed to inhibit the release of IL-1β from RAW 264.7 cells. Similarly, the release of NO was inhibited by AE extract 250 µg/mL and VB 125 µg/mL. This is in agreement with previous reports which indicated that VB inhibited the expression of pro-inflammatory cytokines in immune cells^49–51^. The inhibition occurs when VB binds to the two proinflammatory transcriptional factors, activator protein-1 and NF-κB. This binding results in the downregulation of the two transcription factors^51^ and TGF-beta expression^52^, which affects the synthesis of IL-1β and TNF-α. In addition, another publication indicated that VB inhibits NO synthesis by suppressing iNOS enzyme activity^53^. This mechanism can be used to explain the decrease of IL-1β and NO released from RAW 264.7 cells.

Dermal papilla cells can release several inflammatory cytokines. IL-1α, an inflammatory cytokine, was measured in irradiated dermal papilla cells. Generally, IL-1α is found at the cell membrane surface and is released when the cell membrane is damaged^54,55^. UVA 20 mJ/cm^2^ was used to induce IL-1α expression in dermal papilla cells. Both AE extract and VB effectively suppressed IL-1α release. IL-6 has been recognized as an androgen inducible negative mediator for AGA development^56^. IL-6 is also a proinflammatory mediator, which is upregulated in the hair follicles of AGA patients. There is a report^57^ indicating that hair shaft elongation was inhibited by IL-6 treatment. IL-6 treatment is associated with suppressing the proliferation of matrix cells in cultures of human hair follicles and is known to promote the transition of hair follicles from the anagen to catagen phases in the hair cycle of mice which indicates that shortening the anagen phase results in less time for hair growth.

We induced IL-6 expression by UVB irradiation in the dermal papilla. AE extract 250 µg/mL and VB 125 µg /mL strongly decreased the level of IL-6 released from the irradiated dermal papilla cells. This inhibition could be due to the membrane stabilizing property of VB. We hypothesize that VB forms a stable lipid complex with the cell membrane which may prevent cell membrane damage from UV radiation^58^. These findings suggest that anti-inflammatory, 5α-reductase inhibitory, and dermal papilla growth promoter effects of AE extract were the rational use of AE extract in reducing alopecia. Although AE extract contains many active compounds, we are interested in VB and have used it as the biomarker because VB demonstrates an outstanding effect associated with the anti-hair loss pathway. VB can increase dermal papilla cell proliferation and protection of testosterone-induced dermal papilla cell apoptosis concomitantly with anti-inflammation activity. These activities are an influence effect on the protection of hair loss and promote hair regrowth. VB in the AE extract is possible one component among others that contribute to this benefit. Further study needs to be done to identify other bioactive compounds.

## Conclusion

AE has been used as a traditional medicine in some Asian countries to treat various diseases. AE also has numerous potential therapeutic effects such as being an anti-inflammatory and skin nourishing agent. VB possesses pharmacologically beneficial qualities for human health, including acting as an antioxidant and anti-inflammatory agent. In summary, AE extract and VB manifest positive effects on human dermal papilla and inflammatory conditions that can be used to reduce hair loss and promote hair growth. Our research suggests that AE extract and VB are potential ingredients for future alopecia therapy.

## Materials and methods

### Chemicals

1-(4, 5-Dimethylthiazol-2-yl)-3, 5-diphenylformazan or thiazolyl blue formazan (MTT), finasteride and hydrocortisone were purchased from Sigma-Aldrich (St. Louis, USA). Fetal bovine, 2.5% trypsin serum, penicillin/streptomycin, phosphate buffer solution pH 7.4 were obtained from Gibco (Auckland, New Zealand). Dimethyl sulfoxide or DMSO (Analytical grade) was purchased from Labscan (V.S. Chem House, Thailand). Mouse IL-1β ELISA Instant Kit was purchased from e-bio sciences (Bender-med systems Gmblt, Vienna, Austria). Follicle dermal papilla cell growth medium was purchased from PromoCell (Heidelberg, Germany).

### Plant extraction

AE leaves were purchased from Khaokhoherbary (Phetchaboon, Thailand). The plant collection method and experimental use were in accordance with all the relevant guidelines. The specimens were identified by Assistant Professor Dr. Pranee Nangngam, of the Faculty of Science at Naresuan University. The voucher specimen, collection-number 004163, was deposited at the PNU herbarium also located at the Faculty of Science. The sample leaves of AE were dried at 50 °C for 24 h and then macerated with one or other of the following solvents: 50% ethanol, 95% ethanol, or hexane, and the leaf extracts were allowed to stand for 72 h. Each extract was filtered through Whatman No.1 filter paper before rotary evaporation at 40 °C. The average time for evaporation was 1 h/Liter of extract solution. To make the water extract, leaves of AE were boiled in hot water for 15 min and filtered, and the water was then removed from the extract by freeze-drying. All extracts were stored in vials at -20 °C and protected from light until use.

### HPLC analysis of VB

The HPLC analysis of VB was performed on a Shimadzu HPLC (Bara Scientific, Japan) with a Luna Phenomenex analytical column. (150 mm × 7 mm, 5-µm particle size) (Phenomenex, Deerfield, IL, USA). The mobile phase consisted of 23% ACN (v/v) buffered with 50 mM Sodium Phosphate at pH 2.5. Elution was performed with a flow rate of 1.5 mL/min, detection wavelength at 332 nm, and volume of injection of 20 µL.

### Human dermal papilla cell culture

Human dermal papilla cells (PromoCell, Heidelberg, Germany) were cultured and maintained in a follicle dermal papilla cell growth medium. The growth medium contained growth factors of fetal calf serum 0.04 ml/ml, bovine pituitary extract 0.004 ml/ml, basic fibroblast growth factor recombinant human, 1 ng/ml), and insulin (recombinant human, 5 µg/ml). The cells were incubated under cell culture conditions of 95% relative humidity (RH), 5% CO_2_ and 37 °C. They were sub-cultured until they grew to about 80% confluence.

### Murine macrophage RAW 264.7 cell culture

The RAW 246.7 (ATCC, Virginia, USA), a murine macrophage cell line, was cultured and maintained in DMEM medium (Gibco, Thermo scientific, MA, USA) containing 10% fetal bovine, 10,000 units/ml of penicillin and streptomycin. The cells were incubated under cell culture conditions of 95% RH, 5% CO_2_ and 37 °C. They were sub-cultured until about 80% confluence.

### MTT assay for cell viability and proliferation testing

Ten thousand dermal papilla cells or RAW 264.7 cells were seeded into each well of a 96-well plate. The cells were incubated under cell culture conditions of 95% relative humidity (RH), 5% CO_2_ and 37 °C for 24 h. After incubation, they were treated with AE extract solutions at 0.98 – 500 μg/mL. The incubation time of treatment was 24 h. Fifty micrograms per milliliter of MTT solution were added to each well and the plates incubated for 3 h. The formed formazan crystals were dissolved with 100 µL of DMSO^59^. An absorbance was determined at 595 nm. Relative cell viability was calculated by comparing the treated cells with control cells.

### Cell cycle analysis

The cell cycle was analyzed by a published method^60^ with slight modifications. One hundred thousand dermal papilla cells were seeded into each well of a 6-well plate and incubated under cell culture conditions of 95% relative humidity (RH), 5% CO_2_ and 37 °C for 24 h. After incubation, the cells were treated with plant extract solutions at 31.25 – 500 μg/mL. All cells were collected by trypsinizing and then fixed with 70% cold ethanol for 4 h at -20 °C. The fixed cells were stained using Muse™ cell cycle kit (EMD Millipore Corporation, Hayward, CA), and kept for 30 min protected from light. The cell cycle was analyzed using Guava® easyCyte. The cell cycle profiles, which were represented by DNA contents of each cell cycle phase, were compared among treated cells versus control cells.

### Inhibition effect on testosterone-induced cell apoptosis

The study of dermal papilla cell apoptosis induced by testosterone was slightly modified from the previous report^44^. Ten thousand dermal papilla cells were seeded into each well of a 96-well plate and incubated under cell culture conditions of 95% relative humidity (RH), 5% CO_2_ and 37 °C for 24 h and then pre-treated with 200 µM of testosterone before adding AE extract 250 µg/mL or VB 62.50 µg/mL. The treated cells were incubated for 4 days. On day four, cell viability was determined by MTT assay. The positive control was finasteride at 0.025 µg/mL concentration.

### Determination of 5α-reductase inhibition

Anti-androgenic activity via the steroid 5α-reductase (S5αR) inhibition mechanism was evaluated using a label-free enzymatic inhibitory assay, following the protocol developed by Srivilai and colleagues^38,61,62^. Enzymatic activity was measured by analyzing the DHT formation after enzymatic reaction using liquid chromatography-mass spectrometry (LC–MS).

#### Enzymatic preparation

Androgen-dependent LNCaP cells (CRL-1740™, American Type Culture Collection, VA, USA) provided the source of S5αR. Briefly, the LNCaP cells were cultured in RPMI-1640 medium (Gibco, Paisley, Scotland) supplemented with 10% fetal bovine serum (FBS) and 1% of 10,000 U/mL penicillin G and 10,000 μg/mL streptomycin (Gibco, Paisley, Scotland) at 37 °C under 5% CO_2_ humidified atmosphere before harvesting at ≥ 80% confluent. The protein concentration of homogenized cells was measured using Pierce bicinchoninic acid (BCA) protein assay (Pierce, Rockford, IL, USA). The final total protein was not less than 75 µg in the S5αR inhibitory assay.

#### Enzymatic inhibitory assay

AE extracts were dissolved in DMSO and the inhibition activity determination followed the procedure in Srivilai and colleagues^38,61^. Aliquots of these solutions were added to the assay solution. The enzymatic reaction was composed of test substances, 34.7 µM testosterone, and 1 mM NADPH in Tris buffer pH 7.4. The reaction was started by adding 200 µL of the homogenate enzyme (75 µg total protein). The mixture was incubated at 37 °C for 60 min. The reaction was stopped by adding 300 µL of hydroxylamine (10 mg/mL in 80% (v/v) ethanol) and incubating at 60 °C for 60 min for the derivatization process. The plate was then centrifuged at 1700 g for 10 min and the supernatants were transferred to another plate ready for injection into the LC–MS. C1 and C2 were the two control samples used. Both controls contained the complete reaction mixture as described above but C1 was stopped before enzymatic incubation, whereas, C2 was stopped after 60 min of incubation. The DHT production was measured using LC–MS. The extracted ion chromatogram (EIC) of derivatized DHT (m/z [M + H] + , 306.2428), the area under the curve was used to calculate enzymatic inhibition:

$$\% {\text{Steroid}}\,5\alpha {\text{ - reductase}}\,{\text{inhibition}} = [1 - ({\text{Sample}} - {\text{C}}1)/({\text{C}}2 - {\text{C}}1)] \times 100$$

The standard steroid 5α-reductase inhibitor, finasteride, was used as a positive control.

#### LC–MS method for the measurement of DHT

The determination of DHT was done according to Srivilai and colleagues^38,61^. The Agilent 1260 Infinity Series HPLC system with an auto-sampler accommodating either two 108-vial trays or two 96-well plates (Agilent Technologies, Santa Clara, CA, USA) was used. The analytical reversed-phase column was a Phenomenex Luna® C18 (2) (150 mm × 4.6 mm, 5 µm) with a guard column (Phenomenex C18, 4 mm × 3 mm, 5 µm). The HPLC was connected with an Agilent 6540 UHD Accurate-Mass Q-TOF LC/MS (Agilent Technologies, Santa Clara, CA, USA), equipped with dual electrospray ionization (ESI) in positive mode and m/z range 100–1200. Nitrogen was the nebulizing gas at 30 psi, and the drying gas (10 L/min; 350 °C). The mobile phase was 0.1% (v/v) formic acid in purified water (solvent A) and 0.1% (v/v) formic acid in acetonitrile (LC–MS grade, ACI Labscan, Bangkok, Thailand) as solvent B. The gradient program was used as follows: The initial mobile phase was 60% solvent B and 40% solvent A; solvent B was linearly increased up to 80% over 8 min then held constant for 4 min. Each run was followed by a 2-min post-run. The total run-time analysis was therefore 14 min with the column temperature controlled at 35 °C. The flow rate was 0.5 mL/min and the injection volume was 20 µL. Mass data were analyzed using Agilent Mass Hunter Qualitative Analysis software version B06.00. The data was identified, calculated and presented in term of inhibition activity.

### Determination of IL-1β, NO, and TNF-α secretion inhibition in LPS-stimulated macrophages

One hundred thousand RAW 264.7 cells were seeded into each well of a 24-well plate and incubated under cell culture conditions of 95% relative humidity (RH), 5% CO_2_ and 37 °C for 24 h. Next, the cells were treated with 1 µg/ml lipopolysaccharide (LPS) plus 250 µg/ml AE extracts or 125 µg/ml VB or 62.50 µg/ml VB depending on the tested cytokines. Five micrograms per milliliter of hydrocortisone were the positive control. The treated cells were incubated for 24 h and the supernatants were collected for determination of the amount of IL-1β and TNF-α using an ELISA Kit (Invitrogen, Vienna, Austria). The amount of nitric oxide was determined using the chemical reaction of Griess’s reagent (Sigma-Aldrich, Steinheim, Germany).

### Determination of IL-1α, IL-6 secretion inhibition in UV radiated dermal papilla cells

One hundred thousand dermal papilla cells were seeded into each well of a 24-well plate and incubated under cell culture conditions of 95% relative humidity (RH), 5% CO_2_ and 37 °C for 24 h. The cells were irradiated by 100 mJ/cm^2^ of UVB for the IL-6 study or 20 J/cm^2^ of UVA for IL-1α study using UV irradiation chamber (Opsytec, Dr. Grӧbel GmbH, Ettlingen, Germany). The irradiated cells were immediately treated with 250 µg/ml AE extract or 125 µg/ml VB solution and incubated for 24 h. The expression of IL-1 α and IL-6 in the cell culture supernatants were assayed by an ELISA kit according to the manufacturer's protocol (BioLegend, San Diego, CA; Abcam, Cambridge CB2 OAX, UK).

### Statistical analysis

All data were presented as a mean ± standard deviation (S.D.). Individual differences were evaluated by t-test or one-way ANOVA.
